# Longing for touch and CT-optimal touch perception after interpersonal trauma

**DOI:** 10.1371/journal.pone.0333079

**Published:** 2025-10-27

**Authors:** Birgit Hasenack, Anouk Keizer

**Affiliations:** 1 Faculty of Social and Behavioural Sciences, Experimental Psychology, Utrecht University, Utrecht, The Netherlands; 2 Faculty of Social and Behavioural Sciences, Clinical Psychology, Utrecht University, Utrecht, The Netherlands; Hochschule Niederrhein - Campus Mönchengladbach: Hochschule Niederrhein - Campus Monchengladbach, GERMANY

## Abstract

It has previously been shown that interpersonal trauma (IPT) can negatively impact the experience and perception of social touch. There are, however, also indications that people with IPT might simultaneously experience a stronger longing for touch (LFT). As this has not been systematically investigated, the aim of the current study was to assess the experience of LFT in people with IPT. We additionally assessed if LFT was associated with the perception of CT-optimal touch in this population. In Study 1, 122 women participated in an online experiment. Participants watched and rated videos of CT-optimal (3 cm/s) and CT non-optimal (18 cm/s) touch. They also filled out questionnaires about their LFT and general attitudes towards touch. In Study 2, 44 women participated in a lab-based experiment. Participants were touched by a female researcher with a brush and hand at CT-optimal (3 cm/s) and CT non-optimal (18 cm/s) speeds. They additionally filled out questionnaires about LFT and general attitudes towards touch. In contrast with our hypotheses, women with IPT did not report a stronger LFT or a more negative perception of CT-optimal touch in either study. There was also no significant association between LFT and the perception of touch in women with and without IPT. These non-significant results may be a consequence of the low prevalence of trauma-related psychopathology among participants with IPT in the current study. Implications of these results and suggestions for future research are discussed.

## Introduction

Interpersonal trauma (IPT) can have a profoundly negative effect on general wellbeing. IPT is defined as trauma that develops in response to actions of other people, such as physical or sexual assault [1]. It has repeatedly been shown that these traumatic interpersonal events negatively impact mental health, with victims being at increased risk of developing post-traumatic stress disorder, depression and substance abuse [2–6]. IPT has additionally been associated with poor physical health [7] and interpersonal difficulties [1]. Examples of the latter include insecure attachment [8], emotion dysregulation [9], lower relationship quality [10] and increased distrust [11].

Another interpersonal factor that is affected by IPT is social touch. The neurophysiological processing of social touch has primarily been related to the activation of C-tactile (CT) fibers, which are present in the hairy skin [12]. These fibers project to the posterior insular cortex [13] and respond most optimally to slow (1–10 cm/s) and gentle touch [12]. This touch is therefore also referred to as CT-optimal touch. Although CT-optimal touch is generally perceived to be pleasant [14], it is perceived as less pleasant [15] or even as aversive [16] by people who have experienced IPT. This has been observed for both the physical and vicarious perception of CT-optimal touch [15]. In addition, Strauss and colleagues (2019) found that hippocampal activity is suppressed when people with IPT are touched [16]. They argued that this reflects the suppression of traumatic memories, as interpersonal touch can serve as a trigger (see also [17]). According to Strauss and colleagues (2019), this altered perception may impair the ability to enjoy and seek out interpersonal touch. Consistent with this, reduced intimate touch frequency [18], discomfort with social touch [18,19] and an increased preferred interpersonal distance [20] have been observed in people who have experienced IPT.

Although these results suggest that IPT is primarily negatively associated with the experience and perception of social touch, there are indications that this relation might be more complex. It has been observed in clinical practice that people with IPT do tend to avoid social touch, but simultaneously express a clear desire for it [16]. This ambiguous relation with touch could potentially make people with IPT more vulnerable to the development of longing for touch (LFT), which is the subjective discrepancy between the amount of touch that is desired and received [21]. If touch is avoided but still desired, it can be assumed that this discrepancy will increase. Systematic research into the experience of LFT after IPT is, however, scarce. The only exception is a study by Schellenger (2015). They observed that women who had experienced sexual or physical abuse in adulthood wanted to be touched significantly more by their current romantic partner than control participants [19]. The author did not provide an explanation for this result, so it is unclear if this is indeed a consequence of the aforementioned ambiguous relation with interpersonal touch. In addition, as Schellenger (2015) exclusively focused on romantic relationships, it can be questioned to what extent these findings are representative for the amount of LFT that is experienced in other social relations. This is especially relevant in light of the results by Beβler et al. (2019), who found that touch frequency and the desire for touch were higher in romantic relations than in friendships and contact with acquaintances. Additional research is therefore clearly needed to further investigate the experience of LFT after IPT.

There are several reasons why it is important to understand the relation between IPT and LFT. First, LFT can have negative effects on general wellbeing. It has previously been associated with increased loneliness and stress [22], as well as reduced quality of life [23] and mental health [22]. By understanding to what extent LFT is experienced in people with IPT, we can therefore also determine if they are at increased risk of experiencing these negative consequences. Second, it may provide new insights into the experience and perception of touch after IPT. In non-clinical populations, LFT has been positively associated with the perception of CT-optimal touch [24]. It is unclear if a similar association can be observed in people with IPT, as current research suggest that an aversive perception of touch [16] might be observed alongside an increased LFT [16,19]. Investigating this apparent discrepancy will deepen our understanding of the touch-related consequences of IPT.

The primary aim of the current study is therefore to further investigate the relation between IPT and LFT. The study consist of two sub-studies: in Study 1, we investigated the association between LFT and the vicarious perception of CT-optimal touch in an online experiment. In Study 2, we assessed the relation between LFT and the physical perception of CT-optimal touch in a lab experiment. Only women were included in these studies. This was based on the observation that women may be at an increased risk of experiencing IPT (see, e.g., [25–27]). We additionally wanted to remove potential gender confounds in the perception of interpersonal touch [28]. Based on previous findings, we hypothesized that women with IPT would experience more LFT [16,19]. We also expected that women with IPT would report a more negative vicarious [15] and physical perception of CT-optimal touch [15–16] than women without IPT. For women without IPT, we hypothesized that LFT would be positively associated with the vicarious and physical perception of CT-optimal touch [24]. For women with IPT, we conducted an exploratory analysis on this association due to the absence of previous literature. In both Study 1 and Study 2, we also assessed general attitudes towards touch. As previous studies did not find that people with IPT differed significantly on attitudinal measures [15,29], we did not expect to observe any significant differences between women with and without IPT.

## Study 1

### Method

#### Participants.

Data was collected between November 2023 and July 2024. Due to the scarcity of previous research, sample size was based on the number of participants that could be recruited within this period. A total of 274 people participated in the study. The original dataset also contained male and non-binary participants, which were not included in the current study. These participants (n = 78) and incomplete responses (n = 66) were removed from the dataset. Eight additional participants had to be excluded from further analysis because they filled out the NLETQ incorrectly (n = 6), or because they did not indicate if they had been diagnosed with a psychological disorder (n = 2). No participants had to be removed due to failing the attention check (see *Procedure*). The final sample therefore consisted of 122 participants, of whom 57 had experienced at least one traumatic interpersonal event. Participants with IPT were slightly older (*M* = 22.91; *SD* = 3.58) than participants without IPT (*M* = 21.42, *SD *= 1.90), t(82.51) = −2.827, *p* = .006. We did not include age as a covariate in subsequent analyses, given that all participants were in early adulthood and younger than 36. The presence of self-reported disorders was assessed in each group, as previous research has shown that these can influence the perception of touch (see, e.g., [30–33]). We therefore also assessed the distribution of these disorders in both groups prior to the primary analyses. More participants with IPT had been diagnosed with a psychological disorder (n = 13) than participants without IPT (n = 3), X^^2^^ (1) = 8.820, *p* = .003. Participants with IPT were also more likely to have been diagnosed with a neurological disorder (n = 17) than participants without IPT (n = 4), X^^2^^ (1) = 11.94, *p* < .001. Fisher’s test revealed that there was no difference between the groups with respect to skin-related disorders (n_IPT _= 4; n_NoIPT_ = 1), *p* = .184. Following these results, the presence of psychological and neurological disorders were included as binary covariates in the subsequent analyses.

The average age at which the (first) interpersonal traumatic event occurred was 7.22 (*SD* = 4.81), and the average distress that was experienced was 39.45 (*SD* = 32.38; see *NLETQ* for a complete description of these scales). One participant was excluded from calculating the average distress, as they filled out a number larger than the indicated maximum (100). As the rest of the NLETQ was filled out correctly, we decided to include this participant in the rest of the analyses and desciptives.The median number of traumatic events that had been experienced was 4.0. We decided to not create subgroups based on the age of the first traumatic experience and the total number of traumatic events. This was based on the observation that only 11 participants experienced IPT for the first time during adulthood, and that the majority of participants with IPT had experienced more than one traumatic event (n = 44; 77.19%). Sexual violence (not rape) by a stranger (n = 26), sexual violence (not rape) by a known person (n = 24) were the most commonly reported types of IPT, followed by psychological (emotional) abuse during childhood (n = 19), physical violence by a known person (n = 18), physical violence by a stranger (n = 10), physical abuse during childhood (n = 7), rape by a known person (n = 8) and incest/sexual abuse during childhood (n = 5).

#### Touch deprivation scale.

The Touch Deprivation Scale (TDS; [34]) was used to assess LFT. The TDS consists of 16 items. Responses are provided on a 5-point Likert scale, with responses ranging from 1 (*strongly disagree*) to 5 (*strongly agree*). The TDS has previously been validated [34]. The TDS consists of three subscales: touch deprivation, longing for touch and sex for these. These subscales were analyzed separately in the original study [34] and subsequent research [35,36]. In the current study, only the LFT-subscale was used in the current study. This subscale consists of four items (e.g., “*Some days I long to be held, but have no one to hold me*”). A total score was calculated for this subscale by summing the responses to these four items. Total scores could range between 5 and 20, with higher scores indicating a stronger LFT. The internal consistency of this subscale was high in the current study, Cronbach’s α = .816.

#### Touch experiences and attitudes questionnaire.

Tactile attitudes and experiences were assessed with the Touch Experiences and Attitudes Questionnaire (TEAQ; [29]). The original questionnaire consists of 57 items. However, as longer questionnaires might increase response burden, we used a shorter version of the TEAQ (Trotter et al., 2018). This version contains 37 items that are rated on a 5-point Likert scale (1 = *disagree strongly*; 5 = *agree strongly*). The items are divided into five subscales: attitudes towards familiar touch (AFT), current intimate touch (CIT), childhood touch (ChT), attitude to self-care (ASC), attitude to intimate touch (AIT). As we are only interested in the experience of interpersonal touch, we did not include the ASC subscale in the analyses. An average score was calculated for each other subscale, with higher scores indicating a more positive attitude or experience. In the current study, the reliability of the used subscales was acceptable to excellent (for all, α > .743). It should be noted that the TEAQ-37 has previously only been validated in a Russian subsample. However, it has recently been used as the base for a short version of the TEAQ (TEAQ-S). The TEAQ-S been validated in German, French and English samples [37]. This version was not available at the time of data collection, but given the similarities between the TEAQ-S and the TEAQ-37 we are confident in the use of the latter in the current sample.

#### NLETQ.

The Negative Life Experiences and Trauma Questionnaire (NLETQ; [38] was used to assess interpersonal trauma. The NLETQ consists of 24 items, but only items 3–5 and 9–14 were used in the current study. For each item, participants indicate the frequency with which they have experienced the event, how old they were and how distressing this event was. If the event happened multiple times, they provide the age at which the event was first experienced and indicate how distressing the most severe event was. Distress is indicated on a scale from 0 (*not distressing at all*) to 100 (*very distressing*). While the validity and reliability of the NLETQ have not been assessed, the questionnaire has been used in previous research (see, e.g., [39–41]).

#### Visual touch stimuli.

Two 10 second videos were used to measure vicarious touch perception. The videos depicted a male hand that stroked the forearm of a female with a brush either at a CT-optimal (3 cm/s) or CT non-optimal (18 cm/s) speed. Each video was shown eight times in a randomized order. After each video, participants answered five questions about their perception and experience (1. *How did the videoclip make you feel?* 2. *How do you think the person giving the touch would rate the touch?* 3. *How do you think the person being touched would rate the touch?* 4. *How would you rate the touch?* 5. *How much would you like to be touched like that?*). These questions have been used before by Meijer et al. (2022) [24]. In line with Meijer et al. (2022), an average score was calculated across all questions, with higher scores indicating a more positive perception of the video. A composite score was used to avoid the need for multiple analyses and because the internal consistency of the questionnaire was excellent for both CT-optimal touch (Cronbach’s α = .976) and CT non-optimal touch (Cronbach’s α = .965).

#### Procedure.

All participants provided digital informed consent at the start of the experiment. They first filled out demographic information, after which they were presented with the touch videos. Participants subsequently filled out the NLETQ, TEAQ and TDS. The order of these questionnaires was randomized. The test battery also contained a few additional questionnaires that were not used in the current study. As an attention check, an additional item was incorporated in the TEAQ (“*Please indicate “slightly agree” here*”). Only participants who correctly responded to this item were included in the analyses. Students from Utrecht University received credits for participating in the experiment. Other participants did not receive any compensation. The experiment took around 30 minutes to complete. The study was approved by and executed in accordance with the guidelines of the ethical board of Utrecht University (23–0320). The study was not pre-registered.

#### Data analysis.

Data was analyzed in SPSS (28.0.1.1). An ANCOVA was used to assess differences in LFT between the groups, with the presence of psychological and neurological disorders as binary covariates. A mixed-measures ANCOVA was used to assess the relation between LFT and touch perception. LFT VAS scores were mean-centered prior to this analysis, and the presence of psychological and neurological disorders were used as binary covariates. Four additional Bonferonni-corrected ANCOVAs were used to assess differences on the subscales of the TEAQ, with the presence of psychological and neurological disorders as binary covariates. The assumption of homogeneity of variances was violated for the AIT subscale, F(1,120) = 4.119, *p* = .045. However, given that the variance ratio was below 1.5, robustness of the parametric analysis was assumed [42]. Visual inspection of the Q-Q plots indicated that data on the ChT subscale was skewed for women with and without IPT, and on the CIT subscale for women with IPT. We continued with parametric analyses for these subscales following the central limit theorem. For all analyses α = .05 (two-tailed).

### Results

#### Longing for touch.

Means and standard deviations are reported in Table 1. A univariate ANCOVA was conducted with interpersonal trauma as predictor and psychological disorders and neurological disorders as binary covariates. There was no significant main effect for IPT, F(1, 118) =.207, *p* = .650. The presence of neurological (F(1,118) =.000, *p* = .990) and psychological disorders (F(1,118) = 2.801, *p* = .097) were not significantly associated with LFT.

**Table 1:** Means and standard deviations. Note that the means and standard deviations for the perceived pleasantness of CT-optimal and CT non-optimal touch are based on a smaller sample (see Results).

	*M (SD)*	
	Participants with IPT	Participants without IPT
Perceived pleasantness CT-optimal touch	63.11 (13.79)	60.81 (14.65)
Perceived pleasantness CT non-optimal touch	48.64 (14.37)	43.15 (14.00)
LFT (TDS)	10.09 (4.13)	9.40 (3.77)
Attitude towards familiar touch (TEAQ)	27.21 (4.39)	25.69 (5.51)
Childhood touch (TEAQ)	31.19 (7.60)	33.75 (5.97)
Current intimate touch (TEAQ)	17.51 (5.50)	17.63 (5.01)
Attitude towards intimate touch (TEAQ)	42.95 (4.41)	41.40 (5.28)

#### Longing for touch and vicarious touch perception.

Due to a technical error in the online experiment, 16 participants had to be excluded from analyses involving vicarious touch perception. That meant that the sample for this analysis consisted of 47 women with IPT and 59 women without IPT. As the distribution of psychological and neurological disorders still differed significantly between groups (for both *p* < .015), both variables were included as binary covariates again. A repeated measures ANCOVA revealed that CT-optimal touch (*M* = 61.83, *SD* = 14.25) was rated as significantly more pleasant than CT non-optimal touch (*M* = 45.59, *SD* = 14.36), F(1, 100) = 4.198, *p* = .043, partial-η^^2^^ = .040. There were no significant main effects for LFT, F(1,100) =.013, *p* = .909, IPT, F(1,100) = 3.952, *p* = .050, the presence of psychological disorders, F(1,100) = 1.460, *p* = .230, or the presence of neurological disorders F(1, 100) =.050, *p* = .824. There was no significant interaction between IPT and LFT, F(1,100) =.017, *p* = .897. All other interactions were non-significant as well, for all *p* > .250 (for a complete overview, see Table A in the S1 File).

#### Attitudes towards touch.

Four Bonferonni-corrected ANOVAs were conducted to assess differences in touch-related attitudes and experiences. The entire sample was used for these analyses, and the presence of neurological and psychological disorders were added as binary covariates. There was no significant difference on the AFT, F(1,118) = 2.668, *p* = .105, ChT, F(1,118) = 3.009, *p* = .085, CIT, F(1,118) =.055, *p* = .815 or AIT subscales, F(1,118) = 4.296, *p* = .040. The covariates were not significantly associated with any subscale, for all *p* >. 030.

### Discussion

The aim of Study 1 was to assess LFT and its association with the vicarious perception of CT-optimal touch in women with IPT. The results are only partially in line with our expectations. As hypothesized, we did not observe significant differences between the groups with respect to their attitudes towards touch. This is also consistent with previous research [15,29]. However, in contrast with our hypothesis and previous observations [16,19], women with IPT did not report more LFT than women without IPT. We did also not observe a more negative vicarious perception of CT-optimal touch in women with IPT, which is inconsistent with our hypothesis and previous research [15–16]. There was no significant association between LFT and the vicarious perception of touch in either group. Although we only explored this analysis for women with IPT, we did expect to find a positive association for women without IPT based on Meijer et al. (2022) [24]. In the following paragraphs, we will discuss the unexpected perceptual results as well as the non-significant association between LFT and the perception of CT-optimal touch, as the interpretation of these results informed the design of Study 2. The implications of the other results will be addressed in the general discussion.

In order to understand the non-significant perceptual differences, it is important to consider the type of touch that was depicted in the videos that were used in Study 1. In these videos, participants could see how an arm was stroked with a make-up brush. Strauss et al. (2019) incorporated a similar condition in their physical experiment, and referred to this as impersonal touch [16]. They investigated how participants with IPT responded to this type of touch, and compared it with interpersonal touch (i.e., skin-to-skin contact). For the impersonal touch condition, they found inconsistent results, as participants with IPT did not always respond more negatively to this condition. However, these participants did consistently perceive interpersonal touch as aversive. Strauss et al. (2019) also found that that hippocampal activity was only suppressed in participants with IPT during interpersonal touch, which could indicate that this type of touch is perceived as more threatening or trigger-inducing. While it has not directly been investigated if similar patterns can be observed in the vicarious perception of impersonal and interpersonal touch, Devine et al. (2020) did find that participants with higher levels of IPT responded negatively to videos that depicted skin-to-skin contact [15]. It should be noted, however, that participants in their study responded similarly to physical interpersonal and impersonal touch. There are several methodological differences that could explain why these results contrast with Strauss et al. (2019). One notable difference is that experimenters wore cotton gloves to administer interpersonal touch in the study by Devine et al. (2020), which may be perceived differently from the direct skin-to-skin contact that was described by Strauss et al. (2019). Despite these partially conflicting results, the possibility should be considered that aversive responses in people with IPT might primarily be observed in the context of interpersonal and skin-to-skin touch. In order to further investigate this, both interpersonal and impersonal touch will be included in Study 2.

It is possible that we did not observe a significant association between LFT and touch perception due to the measure that was used to assess LFT in Study 1. Meijer et al. (2022) specifically measured the extent to which LFT was experienced at the time of the experiment. In contrast, the TDS does not assess LFT in relation to a specific point in time (e.g., “*I often wish…*” “*Some days…*”) [24]. It has previously been suggested that perceptual changes related to the deprivation of a need are temporary, and will disappear once the associated need is fulfilled [43]. It might therefore be more difficult to find a significant association with the TDS, as this measure does not necessarily reflect current levels of LFT. We will therefore include the same measure that was used by Meijer et al. (2022) to assess LFT in Study 2.

## Study 2

### Method

#### Participants.

Data was collected between November 2024 and January 2025. Similar to Study 1, sample size was based on the number of participants that could be recruited in this period. A total of 79 women participated in the lab-experiment. Due to a technical error, not all responses were saved for the first 35 participants. These were therefore removed from the dataset. As such, the final sample consisted of 44 participants. All participants were older than 18 years old. Twenty-six participants had experienced IPT. Participants with (*M* = 23.65, *SD* = 6.23) and without IPT (*M* = 21.56, *SD *= 1.34) did not differ with respect to age, t(28.26) = −1.663, *p* =  .107. Similar to Study 1, the presence of self-reported diagnoses was assessed in each group. The distribution of these diagnoses in each group was checked prior to the primary analyses. Fisher’s tests indicated that there was no significant difference in the distribution of psychological (*p* = .451) neurological (*p* = .567) or skin-related disorders (*p* = 1.00) in the two groups. These variables were therefore not included as covariates in the subsequent analyses.

The average age at which the (first) interpersonal traumatic event was experienced was 13.74 (*SD* = 4.61), with the average reported distress being 69.67 (*SD* = 20.54). The median number of traumatic events was 1.5. Similar to Study 1, we did create subgroups based on the age of the first traumatic experience or the number of traumatic events that had been experienced. Only five participants experienced the first traumatic event in adulthood and the majority (n = 22; 84.62%) had experienced more than one event. The most common types of interpersonal trauma were psychological (emotional) abuse during childhood (n = 11) and sexual violence (not rape) by a stranger (n = 10), followed by physical abuse during childhood (n = 8), physical violence by a stranger (n = 7), rape by a stranger (n = 6), rape by a known person (n = 4), incest/sexual abuse during childhood (n = 3) and physical violence by a known person (n = 1).

#### Materials.

Similar to Study 1, the LFT subscale of the TDS (Cronbach’s α = .781) and TEAQ-37 (for all, Cronbach’s α > .773) were included in the experiment. In addition, the LFT VAS [24] was used to assess current levels of LFT. This scale consists of two items (1. “*Currently I would prefer to be touched by others” … 2. “Currently I would prefer to touch others”...).* Participants respond to these items with a visual analogue scale (VAS), that ranges from 0 (“*Less*”) to 100 (*“More*”). In line with our previous studies (see, e.g., [23,24,44], responses on the two items were averaged to calculate an average LFT score. Composite scores have previously been used due to the excellent internal consistency of the questionnaire [23,24,44]. The internal consistency of the scale was acceptable in the current sample, Cronbach’s α = .766.

#### Physical touch stimuli.

Touch stimuli were administered by trained (female) researchers. Touch was administered to the left forearm of the participants. Two 9x4 cm areas were marked on the forearm, to which touch was alternately applied. Touch was administered either by the hand of the researchers or by a make-up brush. This will be hereafter referred as the different modes of touch. Touch was administered at either a CT-optimal (3 cm/s) or CT non-optimal (18 cm/s) speed. Stimuli were presented in two blocks, with one for each mode. The order of the blocks was randomized between participants. Each touch velocity was presented eight times during a block in a randomized order. After each trial participants rated the pleasantness of the touch on a VAS ranging from 0 (“*Very unpleasant”*) to 100 (*“Very pleasant”*).

#### Procedure.

Qualtrics was used to record pleasantness ratings and questionnaire responses. All participants provided digital informed consent at the start of the experiment. After filling out demographic questions, they completed the touch trials. They subsequently filled out the LFT-VAS, TDS and the TEAQ-37. These questionnaires were not presented in a randomized order. The test battery also included other questionnaires that were not used in the current study. Participants either received a small monetary reward or credits for participating in the study. The entire experiment took around 40 minutes to complete. The study was approved by and executed in accordance with the guidelines of the ethical board of Utrecht University (23–0320). The study was not pre-registered.

#### Data analysis.

Data was analyzed in SPSS (28.0.1.1). Two Bonferonni-corrected independent t-tests were used to assess differences in the severity of LFT, with the LFT VAS and TDS as respective outcome measures. Four additional Bonferonni-corrected t-tests were used to assess differences in the attitudes towards touch. Q-Q plots suggested that there were deviations from normality for LFT VAS data in women without IPT and TDS data in women without IPT. Deviations were also observed for the ChT, CIT and AIT subscales. We therefore also conducted non-parametric Mann Whitney-U tests on these variables. Since these yielded the same results (see S2 File), non-normality was not assumed to be a concern [45]. Results of the parametric t-tests are reported here for the sake of interpretability. The relation between LFT and the perception of CT-optimal touch was assessed with two Bonferonni-corrected repeated-measures ANOVAs, with the LFT VAS and the TDS LFT subscale as continuous predictors. The continuous variables were mean-centered prior to the analysis. Inspection of Q-Q plots raised questions about the distribution of the data for CT-optimal touch (administered by hand) for women without IPT. Data were negatively skewed, primarily due to a single datapoint. This datapoint was not an influential (Cook’s distance < 1) or extreme (< 3SD) outlier. ANOVAs have been shown to be robust to deviations from normality, even for relatively extreme deviations in smaller samples [46]. We therefore decided to continue with the parametric tests, but to interpret the results with some caution. For all analyses, α = .05 (two-tailed).

### Results

#### Longing for touch.

**Fig 1:**
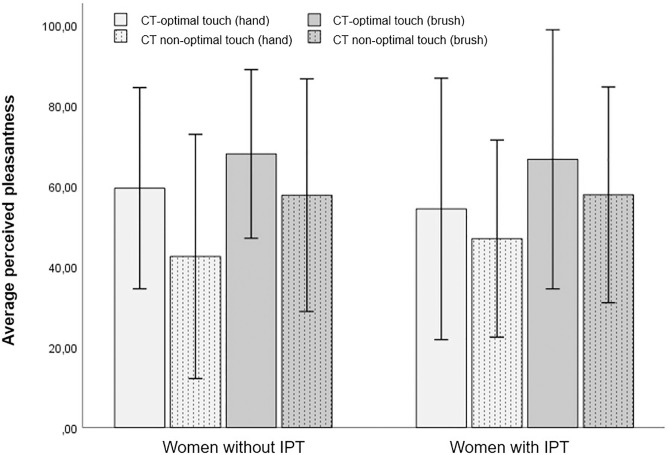
Average perceived pleasantness across conditions in women with and without IPT. Note. Error bars represent the standard deviations (+/- 2SD).

Means and standard deviations are displayed in Table 2 and Fig 1. Two Bonferonni-corrected independent t-tests were conducted to investigate the differences in LFT between participants with and without IPT. Participants without IPT (*M* = 10.11, *SD* = 3.01) did not differ significantly from participants with IPT (*M* = 8.92, *SD* = 3.85) on the TDS LFT subscale, t(42) = 1.097, *p* = .279. The same was observed for the LFT VAS (*M*_*IPT *_= 48.58, *SD*_*IPT*_ = 19.94; *M*_*No IPT *_= 54.58, *SD*_*No IPT*_ = 19.15), t(42) =.998, *p* = .324. These results are in contrast with our hypothesis, as we expected participants with IPT to experience more LFT than participants without IPT.

#### Longing for touch and touch perception.

Two repeated measures ANOVAs were conducted to assess differences in physical touch perception in relation to LFT. In the first analysis, the LFT VAS was used as a continuous predictor. There was a significant main effect for mode, with touch being perceived as more pleasant when it was administered with a brush (*M* = 62.41, *SE* = 1.56) than with a hand (*M* = 50.66, *SE* = 1.49), F(1,40) = 48.795, *p* < .001, partial-η^^2^^ = .550 (see Fig 1). There was also a significant main effect for velocity. In line with our expectations, CT-optimal touch (*M* = 61.93, *SE* = 1.89) was perceived to be more pleasant than CT non-optimal touch (*M* = 51.15, *SE *= 1.95), F(1,40) = 15.722, *p* <. 001, partial-η^^2^^ = .282 (see Fig 1). Contrary to our expectations, there was no significant main effect for LFT VAS scores, F(1,40) = 1.714, *p* = .198, or IPT, F(1, 40) =.000, *p* = .983. LFT was thus not associated with the subjective perception of touch, and women with IPT did not perceive touch more negatively than women without IPT. The interaction between LFT VAS scores and IPT was non-significant as well F(1,40) = 1.705, *p* = .199. We do want to note that inspection of the means (see Table 2 and Fig 1) initially suggested that the difference between CT-optimal and CT non-optimal touch (administered by hand) might be larger for women without IPT than for women with IPT. This would suggest that women with IPT are less sensitive to changes in the velocity of interpersonal touch. However, the associated interaction between mode, velocity and group was non-significant after applying corrections, F(1,40) = 5.315, *p* = .026. None of the remaining interactions were significant either, for all *p* > .030 (for a complete overview, see Table B in the S1 File).

**Table 2:** Means and standard deviations.

	*M (SD)*	
	Participants with IPT	Participants without IPT
Perceived pleasantness CT-optimal touch (hand)	54.25 (16.21)	59.38 (12.48)
Perceived pleasantness CT non-optimal touch (hand)	46.88 (12.22)	42.45 (15.14)
Perceived pleasantness CT-optimal touch (brush)	66.55 (16.06)	67.88 (10.46)
Perceived pleasantness CT non-optimal touch (brush)	57.74 (13.38)	57.65 (14.42)
LFT (VAS)	48.58 (19.94)	54.58 (19.15)
LFT (TDS)	8.92 (3.85)	10.11 (3.01)
Attitude towards familiar touch (TEAQ)	24.19 (5.34)	25.33 (5.46)
Childhood touch (TEAQ)	28.50 (8.50)	31.83 (5.86)
Current intimate touch (TEAQ)	17.46 (5.45)	13.56 (5.44)
Attitude towards intimate touch (TEAQ)	40.42 (6.12)	41.39 (5.21)

In the second analysis, the TDS LFT subscale was used as a continuous predictor. Similar to the first analyses, there were significant main effects for mode F(1,40) = 45.292, *p* < .001, partial-η^^2^^ = .531, and velocity, F(1,40) = 15.697, *p* < .001, partial-η^^2^^ = .282. There were also no significant main effects for TDS scores F(1,40) =.004, *p* = .952 or IPT, F(1, 40) =.016, *p* = 899. These results are in contrast with our hypotheses. In addition, there was no significant interaction between TDS scores and IPT, F(1,40) = 5.128, *p* = .029. All remaining interactions were non-significant as well, for all *p* >. 040 (for a complete overview, see Table C in the S1 File).

#### Attitudes towards touch.

Four Bonferonni-corrected independent t-tests were conducted to investigate differences in touch attitudes between the groups. There was no significant difference between the groups on the AFT, t(42) =.691, *p* = .493, ChT, t(42) = 1.539, *p* = .131, CIT, t(42) = −2.340, *p* = .024 or the AIT, t(42) =.546, *p* =. 588 subscales.

### Discussion

The aim of Study 2 was to further assess LFT and its association with the physical perception of CT-optimal touch in women with IPT. The results only partially support our hypotheses. As expected, and in line with previous research [15,29], no significant attitudinal differences were observed between the groups. However, women with IPT did not report higher levels of LFT than women without IPT. The groups did also not differ with respect to the pleasantness perception of CT-optimal touch, regardless of whether the touch was administered with a brush or a hand. These results are in inconsistent with our hypotheses and previous observations [15,16,19]. It is especially unexpected that women with IPT did not respond more negatively to skin-to-skin touch. We included this condition because Strauss et al. (2019) specifically observed that people with IPT responded consistently aversive to this type of touch [16]. Neither general nor current levels of LFT were significantly associated with the pleasantness perception of touch in either group, which is inconsistent with our hypothesis and the observations by Meijer et al. (2022) [24].

The interpretation and implications of these results will be addressed in the general discussion, but we do want to highlight an unexpected perceptual finding here. We observed that, in general, participants perceived touch that was administered with a brush to be significantly more pleasant than skin-to-skin contact. This does not only contrast with Strauss et al. (2019) and Devine et al. (2020), who did not report a similar main effect [15,16], but also with other studies that examined differences between these types of touch in non-clinical populations. In these studies, either no significant differences were found [47] or a preference for skin-to-skin touch [48]. Similarly to the current study, participants were touched by an unknown experimenter in all aforementioned studies [15,16,47,48]. It therefore seems less likely that the significant difference that we observed is caused by a general discomfort with receiving skin-to skin-to-skin touch from a stranger. An alternative explanation relates to the fact that we did not regulate the texture and temperature of the skin-to-skin touch. Consequentially, we do not know to what extent this varied between trials and experimenters, and how it compared to the with a brush. Thermal variability could especially have influenced how skin-to-skin touch was perceived, given that CT-fibers are sensitive to temperature [49]. Almost all of the aforementioned studies therefore took certain regulatory precautions, such as making the experimenters wear cotton gloves [15] or letting them rub their hands before administering the touch [16,48]. As we did not take such precautions in the current study, we cannot exclude the possibility that the preference for touch that was administered with a brush was primarily driven by textural and thermal differences. It is therefore important that the texture and temperature of both impersonal and interpersonal touch is regulated in future studies.

### General discussion

The aim of the current study was to investigate the extent to which women with IPT experience LFT. We additionally assessed how LFT was related to the vicarious and physical perception of CT-optimal touch in these women. The results of Study 1 and Study 2 are only partially in line with our hypotheses. As has been observed in previous studies [15,29], women with IPT did not differ significantly from women without IPT with respect to their attitudes towards touch. However, in contrast with our hypothesis, women with IPT did not report higher levels of LFT than women without IPT. This is inconsistent with previous clinical observations [16] and research by Schellenger (2015) [19]. In addition, women with IPT did not report a more negative vicarious or physical perception of CT-optimal touch, which is not in line with our expectations and previous studies [15,19]. Current and general levels of LFT were not associated with the pleasantness perception of CT-optimal touch in either group. Although we did not formulate specific hypotheses for this association in women with IPT, we did expect to find a positive association for women without IPT based on the findings by Meijer et al. (2022) [24].

It is unexpected that we did not observe any significant perceptual differences between the groups. As mentioned before, the use of impersonal touch could explain why the results of Study 1 were insignificant (for a full explanation, see Discussion Study 1). However, women with IPT also did not perceive interpersonal touch more negatively in Study 2. It is therefore important to consider alternative explanations as well, such as the potential influence of sample composition. The prevalence of self-reported psychopathology among women with IPT was relatively low in Study 1 (22.8%) and Study 2 (15.4%). In addition, it is unclear if the reported diagnoses were specifically related to the IPT that participants had experienced. This information is relevant, as previous studies have shown that the presence of (trauma-related) psychopathology might mediate the relation between IPT and subsequent negative consequences. This mediation has not only been observed for physical health [7], interpersonal and emotional problems [1], but also for touch behaviours [50]. Examples of the latter include cuddling, hugging and kissing [50]. It has not been directly investigated if changes in the perception of interpersonal touch are similarly mediated by trauma-related psychopathology. However, previous studies that reported significant differences in the perception of touch after IPT either specifically focused on participants with PTSD [16] or participants with high levels of PTSD-symptomatology [20]. This clearly contrasts with the sample composition of the current study: only a small percentage of participants with IPT had been diagnosed with a psychological disorder. Devine et al. (2020) also observed significant perceptual differences, but did not report the presence of trauma-related psychopathology in their sample [15]. Their results can therefore not be directly compared to the current study, but it is possible that the prevalence of psychopathology was higher in these samples. Additional research is necessary to determine if changes in the perception of touch can indeed only be observed in the context of trauma-related psychopathology. To this end, future studies should also consistently report the levels of psychopathology in their samples. This will make it easier to compare and understand potentially conflicting results.

The low prevalence of psychopathology may additionally explain why we did not observe significant differences with respect to LFT. As few studies have systematically assessed the experience of LFT after IPT, we primarily based our hypothesis on the clinical observations of Strauss et al. (2019) [16]. They described that patients with IPT tend to avoid touch, but still express a clear longing for it. We hypothesized that this ambiguous relation with touch could increase the risk of developing LFT. However, Strauss et al. (2019) specifically observed this relation in a clinical setting where patients received treatment for trauma-related psychopathology. The psychological functioning of these patients may therefore not be comparable to that of the participants in the current study, especially because the low prevalence of psychopathology makes it unlikely that many of our participants were receiving psychological treatment at the time of the study. We additionally based our hypothesis on Schellenger (2015), who found that women who had experienced IPT in adulthood reported a stronger LFT in current romantic relationships [19]. As they did not report the prevalence of trauma-related psychopathology in their sample, it is unclear if this differed from the current study and, consequently, if it can explain the inconsistent results. It should additionally be noted that Schellenger (2015) exclusively focused on romantic relationships and IPT in adulthood. In contrast, we assessed a more general sense of LFT and most of our participants had experienced IPT for the first time in childhood. These methodological differences could therefore equally have contributed to the inconsistent results. Future studies should therefore not just investigate if increased LFT is specifically associated with trauma-related psychopathology, but also if it can only be observed in certain social relationships and if it depends on the age at which IPT was experienced.

The composition of the current sample does, however, not explain why LFT was not significantly associated with the vicarious or physical perception of CT-optimal touch. The analyses were conducted exploratively for women with IPT, but we at least expected to find a positive association for women without IPT based on Meijer et al. (2022) [24]. As mentioned before, the non-significant results of Study 1 could potentially be explained by differences in the assessment of LFT. In Study 1, we only assessed general levels of LFT with the TDS. Meijer et al. (2022), on the other hand, used a VAS to assess to what extent participants experienced LFT at the time of the study (for a full discussion, see *Discussion Study 1*). We therefore decided to include this measure in Study 2, but still failed to observe a significant association. The general design of Study 2 did differ from Meijer et al. (2022), with the most notable difference being that we focused on the physical perception of CT-optimal touch rather than the vicarious perception. However, this methodological difference might not necessarily provide an explanation for the conflicting results, since the physical and vicarious perception of CT-optimal touch have been shown to elicit a similar neural response [51]. A potentially more relevant difference is the sample size, which was considerably larger (n = 1982) in the study by Meijer et al. (2022). The regression coefficients that were reported in their study were also relatively small. If the true effect of LFT is small, then it is possible that associations with the perception CT-optimal touch will not be detected in smaller samples [52]. It can then also be questioned how much this association actually contributes to individual differences in the perception of CT-optimal touch in practice.

The only findings that are seemingly consistent with previous observations are the non-significant attitudinal differences between the two groups. Similar results have been reported by Trotter et al. (2018) [29] and Devine et al. (2020) [15]. However, it is important to interpret these findings in the context of their respective studies. In the current study, it is possible that the non-significant differences are merely another consequence of the low levels of trauma-related psychopathology. Trotter et al. (2018), on the other hand, noted that the prevalence of IPT was relatively low in their sample. This might not only have contributed to their non-significant findings, but also limits the generalizability of their results. Devine et al. (2020) did recruit a sample with a high prevalence of childhood IPT, but they observed the non-significant attitudinal differences alongside significant perceptual differences. The authors did not provide an explanation for this discrepancy in their findings. It is possible that actual tactile stimuli are perceived as more threatening than questions about touch, especially since touch was administered by a stranger and the used questionnaire pertained to attitudes towards familial and intimate touch. As such, although the results of the aforementioned studies appear to be consistent, their interpretation might actually be substantially different. It should also be noted that neither Trotter et al. (2019) nor Devine et al. (2020) reported the prevalence of trauma-related psychopathology in their sample, which makes it additionally difficult to determine to what extent these results can be compared to the current study.

The current results also need to be discussed in light of one final methodological limitation. We used the NLETQ to assess the presence of IPT in both studies. Although this questionnaire contains useful items about the frequency and intensity of the experienced traumatic events, it does not actually assess to what extent participants are *currently* affected by these events. As the majority of the participants in the current study experienced IPT during childhood, it is possible that they already had some opportunities to find support and process the traumatic events. This could subsequently have influenced the current results, although we cannot definitely conclude this. Future studies should therefore determine the current impact of IPT and control for this in subsequent analyses.

In conclusion, women with IPT did not significantly from women without IPT with respect to LFT, the vicarious and physical perception of CT-optimal touch, and general attitudes towards touch. These results support the notion that the relation between traumatic interpersonal events and the experience of touch is complex, rather than unequivocally negative. Additional research is needed to further investigate the complexity of this relation. Future studies should especially focus on the influence of potentially mediating factors, such as the presence of trauma-related psychopathology. These studies will provide valuable new insights into the experience and perception of touch after IPT.

## Supporting information

S1 File Supplementary materials. (DOCX)

S2 File Data and analyses. (ZIP)
