# Supplementary material for: Longing for touch and CT-optimal touch perception after interpersonal trauma
Source: PLoS One. 2025 Oct 27;20(10):e0333079. doi: 10.1371/journal.pone.0333079 (PMC12558537; doi:10.1371/journal.pone.0333079)
Supplement: S1 File — (DOCX) [file pone.0333079.s001.docx]

**Supplementary materials to**

Longing for touch and CT-optimal touch perception after interpersonal trauma

Birgit Hasenack^a,b^, Anouk Keizer^a^

^a^ Faculty of Social and Behavioural Sciences, Experimental Psychology, Utrecht University, Utrecht, The Netherlands.

^b^ Faculty of Social and Behavioural Sciences, Clinical Psychology, Utrecht University, Utrecht, The Netherlands. ^*^ Corresponding author at: Faculty of Social and Behavioural Sciences, Utrecht University, Heidelberglaan 1, 3584 CS, Utrecht, the Netherlands. *Email address:* [b.hasenack@uu.nl](mailto:b.hasenack@uu.nl)

Study 1

|  | F (df1, df2) | *p* | partial-η^2^ |
| --- | --- | --- | --- |
| Velocity | 4.198 (1,100) | .043 | .040 |
| IPT | 3.952 (1,100) | .050 | - |
| LFT (TDS subscale) | .013 (1,100) | .909 | - |
| Psychological disorders | 1.460 (1,100) | .230 | - |
| Neurological disorders | .050 (1,100) | .824 | - |
| IPT * LFT | .017 (1,100) | .897 | - |
| Velocity * LFT | .464 (1,100) | .497 |  |
| Velocity * IPT | .526 (1,100) | .470 | - |
| Velocity * psychological disorders | .121 (1,100) | .729 | - |
| Velocity * neurological disorders | 1.327 (1,100) | .252 | - |
| Velocity * IPT * LFT | .598 (1,100) | .441 |  |

**Table A.** *Results of the mixed-measures ANCOVA with the TDS LFT subscale as continuous predictor.*

Study 2

*Non-parametric tests*

Bonferonni-corrected Mann Whitney-U tests indicated that there was no difference between women with (*Mdn* = 8.00) and women without IPT (*Mdn* = 10.00) with respect to TDS scores, U = 174.00, z = -1.44, *p* = .149. LFT VAS scores did also not differ significantly between women with (Mdn = 50.25) and women without IPT (*Mdn* = 58.50), U = 191.50, z = -1.015, *p* = .310.

Bonferonni-corrected Mann Whitney-U tests were also used to analyze differences between the groups on the CIT, AIT and ChT subscales of the TEAQ. There were no differences between women with (*Mdn_CIT_* = 18.50, *Mdn_AIT_* = 41.50, *Mdn_ChT_* = 30.00) and women without IPT (*Mdn_CIT_* = 13.50, *Mdn_AIT_* = 43.50, *Mdn_ChT_* = 31.50) with respect to CIT (U = 327.00, z = 2.23, *p* = .026), AIT (U = 213.50, z = -0.49, *p* = .623 and ChT (U = 184.50, z = -1.184, *p* = .236).

|  | F (df1, df2) | *p* | partial-η^2^ |
| --- | --- | --- | --- |
| Velocity | 15.722(1,40) | < .001 | .282 |
| Mode | 48.795 (1, 40) | < .001 | .550 |
| IPT | .000 (1, 40) | .983 | - |
| LFT (LFT VAS) | 1.714 (1, 40) | .198 | - |
| IPT * LFT | 1.705 (1, 40) | .199 | - |
| Velocity * LFT | .119 (1, 40) | .732 |  |
| Velocity * IPT | .882 (1, 40) | .353 | - |
| Mode * LFT | .056 (1, 40) | .814 | - |
| Mode * IPT | .004 (1, 40) | .953 | - |
| Mode * velocity | 2.236 (1, 40) | .143 |  |
| Velocity * IPT * LFT | 1.691 (1, 40) | .732 |  |
| Mode * IPT * LFT | 4.875 (1, 40) | .033 |  |
| Mode * velocity * IPT | 5.315 (1, 40) | .026 |  |
| Mode * velocity * LFT | 2.489 (1, 40) | .123 |  |
| Velocity * Mode * IPT * LFT | .809 (1, 40) | .374 |  |

**Table B.** *Results of the mixed-measures ANCOVA with the LFT VAS as continuous predictor.*

|  | F (df1, df2) | *p* | partial-η^2^ |
| --- | --- | --- | --- |
| Velocity | 15.697 (1,40) | < .001 | .282 |
| Mode | 45.292 (1, 40) | < .001 | .531 |
| IPT | .016 (1, 40) | .899 | - |
| LFT (TDS subscale) | .004 (1, 40) | .952 | - |
| IPT * LFT | 5.128 (1, 40) | .029 | - |
| Velocity * LFT | .127 (1, 40) | .724 |  |
| Velocity * IPT | .858 (1, 40) | .360 | - |
| Mode * LFT | .236 (1, 40) | .630 | - |
| Mode * IPT | .001 (1, 40) | .975 | - |
| Mode * velocity | 2.237 (1, 40) | .143 |  |
| Velocity * IPT * LFT | 1.427 (1, 40) | .239 |  |
| Mode * IPT * LFT | .471 (1, 40) | .496 |  |
| Mode * velocity * IPT | 4.455 (1, 40) | .041 |  |
| Mode * velocity * LFT | .247 (1, 40) | .622 |  |
| Velocity * Mode * IPT * LFT | 1.221 (1, 40) | .276 |  |

**Table C.** *Results of the mixed-measures ANCOVA with the TDS LFT subscale as continuous predictor.*
